# A Novel Korean Red Ginseng Compound Gintonin Inhibited Inflammation by MAPK and NF-*κ*B Pathways and Recovered the Levels of mir-34a and mir-93 in RAW 264.7 Cells

**DOI:** 10.1155/2015/624132

**Published:** 2015-10-12

**Authors:** Evelyn Saba, Bo Ra Jeon, Da-Hye Jeong, Kija Lee, Youn-Kyoung Goo, Dongmi Kwak, Suk Kim, Seong-Soo Roh, Sung Dae Kim, Seung-Yeol Nah, Man Hee Rhee

**Affiliations:** ^1^Laboratory of Veterinary Physiology & Cell Signaling, College of Veterinary Medicine, Kyungpook National University, Daegu 41566, Republic of Korea; ^2^Laboratory of Medical Imaging, College of Veterinary Medicine, Kyungpook National University, Daegu 41566, Republic of Korea; ^3^Department of Parasitology and Tropical Medicine, Kyungpook National University School of Medicine, Daegu 41944, Republic of Korea; ^4^Laboratory of Parasitology, College of Veterinary Medicine, Kyungpook National University, Daegu 41566, Republic of Korea; ^5^Institute of Agriculture and Life Science, Gyeongsang National University, Jinju 52828, Republic of Korea; ^6^College of Korean Medicine, Daegu Haany University, Daegu 42158, Republic of Korea; ^7^Research Center, Dongnam Institute of Radiological and Medical Sciences, Busan 46033, Republic of Korea; ^8^Department of Physiology, College of Veterinary Medicine and BioMolecular Informatics Center, Konkuk University, Seoul 05029, Republic of Korea

## Abstract

The beneficial health promoting effects of ginseng from vitalizing the body to enhancing long life have been well explored very rapidly in the past few years. Up till now many ginsenosides have been discovered for their marvelous therapeutic effects. However during past three years, a novel ginseng compound has been discovered, called gintonin, that differs from other ginsenosides on the basis of its signal transduction and chemical nature. Gintonin has been widely studied for its anti-Alzheimer's disease activities and other neuropathies. However, its anti-inflammatory activity remained unexplored. In our study we have reported for the first time the anti-inflammatory activity of gintonin on RAW 264.7 cells. We found that gintonin potently suppresses the nitric oxide production without any cytotoxicity at given doses and also efficiently suppressed the levels of proinflammatory cytokines. Moreover, it mediaes its signal transduction via MAPK and NF-*κ*B pathways and revives the levels of mir-34a and mir-93. These findings are valuable for the anti-inflammatory effects of this new compound with particular reference to microRNA involvement in the ginseng family.

## 1. Introduction

Toll-like receptors activation occurs when invading pathogens and microbes attach to them which in turn terminate to inflammatory pathways. The kind of pathway that is to be activated by a pathogen depends on the type of pathogen associated molecular patterns (PAMPs) which are specifically recognized by toll-like receptors (TLRs) [1–4]. There are 6 kinds of toll-like receptors; for example, TLR2 identifies lipopeptides, TLR4 recognizes lipopolysaccharides (LPS), and TLRs 3, 7, 8, and 9 are responsible for the signaling of microbial nucleic acids. The association of lipopolysaccharides to TLR4 terminates into the activation of inflammatory cascade leading to the release of proinflammatory cytokines like COX-2, iNOS, TNF-*α*, IL-1*β*, and IL-6. These proinflammatory cytokines are the primary factors for initiation of inflammatory process which progress to the activation of pathways like MAPK, NF-*κ*B, and PI3K/AKT. The NF-*κ*B pathway is the classical pathway for inflammation. It consists of IKK ubiquitination which causes the disassociation of IKB/*α* due to which nuclear translocation of NF-*κ*B occurs and proinflammatory cytokines are released. In case of MAPK pathway also there are major factors like JNK, ERK, and p38 whose activation causes the translocation of Activator Protein-1 (AP-1) gene inside nucleus causing the intensified inflammatory response [5, 6].

Panax Ginseng Meyer (CA) is a herbal tonic being widely used in countries like China, Japan, and Korea for its beneficial effects on health. Ginseng all over the world is mostly known for its marvelous effects in enhancing reproductive health and antiageing properties. Ginseng in a broader classification consists of saponin fraction also called as ginsenosides and nonsaponin fractions. Ginsenosides are the major compound of ginseng that has been studied and researched extensively for pharmacologic and therapeutic effects [7]. Gintonin which is being classified as a nonsaponin fraction had been discovered recently since 3 years and is found to be different from other ginsenosides on the basis of its signal transduction properties via LPA receptors and mobilization of calcium channels in xenopus oocytes at a low concentration [8–10]. Chemically gintonin consists of large amounts of amino acids, the major being phenylalanine. In case of carbohydrates, glucose is the major component of gintonin. Among the lipid components, it is linoleic acid that is most abundantly found in gintonin. So gintonin is actually a glycolipoprotein component of ginseng [11].

MicroRNAs which are being recently explored are a topic of interest for scientific communities these days. Up till now these small 21-22 nucleotide containing noncoding small RNAs have been studied for almost all the bodily functions. They work by either silencing or overexpressing the target RNA for the proteins of interest [12–14]. In this manner they influence every pathological conditions in cells. In case of anti-inflammation area, many MicroRNAs are discovered and explored but in our study we selected micro-RNA 34a and micro-RNA 93. These two MicroRNAs are relatively new to inflammation and there is only limited data on this aspect.

So here in our investigation, we reported for the first time relatively two different MicroRNAs to be explored in a natural compound like gintonin with its anti-inflammatory effects in terms of nitric oxide production, suppression of proinflammatory cytokines, and signal transduction via NF-*κ*B and MAPK pathways.

## 2. Materials and Methods

### 2.1. Gintonin Preparation

Four-year-old ginseng roots approximately 1 kilogram were grounded into small pieces and refluxed with ethanol (EtOH) 80% for 8 h at 80°C for 3 consecutive times. These EtOH extracts were then dried and concentrated. The remaining steps were according to the fractionation by Choi et al. [15].

### 2.2. Materials

Dulbecco's modified Eagle's medium (DMEM) (Daegu, Korea), fetal bovine serum (FBS) (WelGene Co., Korea), streptomycin and penicillin (Lonza, MD, USA), TRIZOL reagent (Invitrogen, Carlsbad, CA, USA), oligodT (Bioneer Oligo Synthesis), and iNOS, COX-2, TNF-*α*, IL-6, and IL-1*β* primers were purchased from Bioneer; LPS (*Escherichia coli* 055:B5) and 3-(4,5-dimethylthiazol-2-yl)-2,5-diphenyltetrazolium bromide (MTT) were obtained from Sigma Aldrich. Specific antibodies that were used against phospho- and total form of ERK, JNK, p38, IKK *α*/*β*, I*κ*B, NF*κ*B p65, iNOS, COX-2, *β*-actin, and secondary antibody rabbit HRP linked antibody were purchased from Cell Signaling Technology. All other reagents and chemicals were obtained from Sigma Aldrich.

### 2.3. Cell Culture

Murine macrophage cell line RAW 264.7 cells, obtained from American Type culture collection, were cultured in Dulbecco's modified Eagle's medium (DMEM) supplemented with 8% fetal bovine serum (FBS) (WelGene Co., Daejeon) and 100 IU/mL penicillin and 100 *μ*g/mL streptomycin sulfate (Lonza, MD, USA). The incubating conditions were maintained at humidified 5% CO_2_ at 37°C.

### 2.4. Nitric Oxide (NO) Assay

Griess reaction assay was the basis for determining the nitric oxide production. Briefly, RAW 264.7 cells were seeded in 96-well plate and incubated with or without LPS (0.1 *μ*g/mL) in absence or presence of gintonin at indicated concentrations for 18 h. The cell culture supernatants (100 *μ*L) were thoroughly mixed with Griess reagent (0.2% naphthylethylenediamine dihydrochloride and 2% sulphanilamide in 5% phosphoric acid) in DDW at equal volumes and then incubated for 5 min at room temperature. The absorbance in each well was then measured at 540 nm in microplate reader (Versamax).

### 2.5. Cell Viability (MTT) Assay

Cell viability assay was done to access the cytotoxicity in cells using 3-(4,5-dimethylthiazol-2-yl)-2,5-diphenyltetrazolium bromide reagent which was added to culture medium at a final concentration of 0.1 mg/mL. After 4 h of incubation at 37°C in 5% CO_2_, the resulting violet colored crystals were dissolved in dimethyl sulfoxide (DMSO) 100 *μ*L/well and absorbance values were measured at 560 nm.

### 2.6. RNA Extraction and qRT-PCR

For total RNA extraction, RAW 264.7 cells were pretreated with or without gintonin at indicated concentrations for 30 min and then stimulated with LPS (0.1 *μ*g/mL) for 18 h. RNA was extracted using TRIZOL reagent (Invitrogen, Carlsbad, CA, USA) following the manufacturer's instructions. Total RNA (2 *μ*g) was annealed with OligodT (Bioneer Co., Daejeon) for 10 min at 70°C and cooled for 5 min on ice, reverse transcribed using reverse transcriptase premix (Bioneer Co., Daejeon) in 20 *μ*L of reaction mixture, and run for 90 min at 42.5°C using thermal cycler. The reactions were then terminated at 95°C for 5 min to inactivate the reverse transcriptase. The reverse transcription polymerase chain reaction was performed using aliquots of cDNA obtained from RT reaction in a PCR premix (Bioneer Co., Daejeon). The PCR products were afterwards electrophorised on 1% agarose gel stained with ethidium bromide and visualized using Eagle Eyes image analysis software (Stratagene, La Jolla, CA). The intensity of band densities was normalized for corresponding GAPDH, which is housekeeping gene used as an RNA internal standard and ratios were compared. Quantitative PCR primers sequence is given in Table 1.

### 2.7. Western Blot Analysis

RAW 264.7 cells were treated or left untreated with gintonin (6.25–50 *μ*g/mL) in the presence or absence of LPS (0.1 *μ*g/mL). Cytosolic and nuclear proteins were extracted according to the instructions of NE-PER nuclear and cytosolic extraction reagents (Thermo Scientific, #78833 and #78835). Proteins were then measured using PROMEASURE assay kit (PRO-PREP, iNtRON Biotechnology). Then they were separated by 10% SDS-PAGE and transferred onto PVDF (Millipore, Immobilon-P, Billerica MA, USA). Nonspecific binding on the nitrocellulose filter paper was minimized using blocking buffer containing 5% nonfat dry milk and 0.1% Tween-20 in TBS. The membranes were then incubated with specific primary antibodies overnight at 4°C followed by 1 h incubation with horseradish peroxidase-conjugated anti-rabbit antibody (1 : 3000 dilution, Cell signaling). Bound antibodies were visualized using enhanced chemiluminescence (Supex) and images were analyzed using ImageJ software. *β*-actin was taken as internal control.

### 2.8. Transient Transfection and Luciferase Assay

For measuring luciferase activity, HEK 293T cells were cultured in 60 mm well plates for 24 h and then transfected with TK Renilla (pRL-TK) and NF-*κ*B firefly luciferase (pNF-*κ*B-Luc) constructs according to Calcium-Phosphate method. Six h after transfection the media were changed to normal DMEM supplemented with FBS and penicillin streptomycin antibiotic and then again incubated for 18 h. After incubation, cells were seeded in 24-well plates and after 18 h incubation again, they were pretreated with gintonin (6.25–50 *μ*g/mL) for 30 min before PMA stimulation for 6 h. Next cells were quantified for luciferase activity using Promega Dual-Glo luciferase assay kit according to manufacturer's instructions. GloMax luminometer (Promega) was used for measuring luciferase activity. Luciferase activity was normalized to TK Renilla. The similar protocol was followed for the expression of AP-1 transcription factor.

### 2.9. Real-Time PCR

For microRNA expression analysis, RAW 264.7 cells were seeded in 6-well plates and pretreated with gintonin (12.5–50 *μ*g/mL) 30 min before LPS treatment (5 *μ*g/mL). Total RNA was extracted from cells using the miRNeasy Mini Kit (Qiagen). RNA then underwent reverse transcription using the miScript RT II kit (Qiagen). All gene transcripts were measured by real-time PCR with the SYBR Green Master Mix using miScript SYBR (Qiagen). The primer assays for real-time PCR analysis are as follows: Mm_miR-34a_1, Mm_miR-93_1, and Hs_RNU6-2_11 (Qiagen). RNU6 was taken as internal control.

### 2.10. Statistical Analysis

All of the data is presented as mean ± SEM. One way ANOVA and Dunnett's test were applied for the statistical evaluation of data. Statistical analyses with *P* < 0.01 were considered significant.

## 3. Results

### 3.1. Suppression of Inflammation Induced by LPS by Gintonin in a Dose Dependent Manner

**Figure 1:**
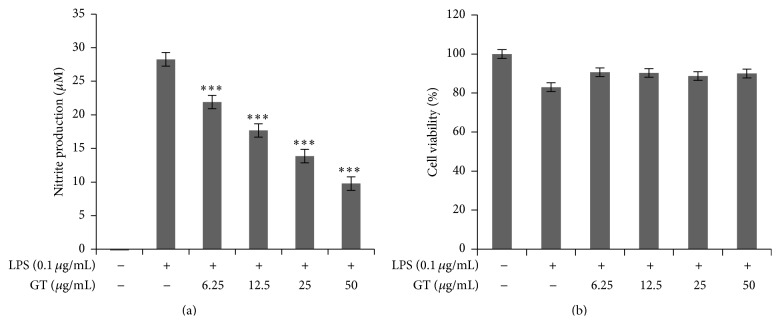
Gintonin inhibited LPS mediated NO release without any cytotoxicity. RAW 264.7 cells were seeded in 96-well plate for 18 h and then preincubated with gintonin (6.25 *μ*g/mL–50 *μ*g/mL) for 30 min and then stimulated with LPS (0.1 *μ*g/mL) for 18 h. The cell supernatant was then transferred to a 96-well plate and mixed with equal amounts of Griess reagent, and then NO production was measured at 540 nm (a). Effects of gintonin on cell viability were measured by MTT assay and absorbance was measured at 560 nm (b). Values in bar graph are mean ± SEM of at least 4 independent experiments. ^*∗∗∗*^ *P* < 0.001 compared to LPS only.

NO production is stimulated by the endotoxin LPS. In addition to this LPS acts as immunostimulatory factor for Gram-negative bacteria and can cause systemic inflammation syndrome if excessively stimulated. For this reason, we investigated whether gintonin affects LPS induced NO production in RAW 264.7 cells. As has been shown in Figure 1(a), gintonin sharply attenuated LPS evoked NO production when it was injected to culture media 30 min after the sample injection. Moreover, it did not show any cytotoxic effect over this concentration range in study as depicted in Figure 1(b).

### 3.2. Attenuation in the Levels of Proinflammatory Cytokines by Gintonin in RAW 264.7 Cells

**Figure 2:**
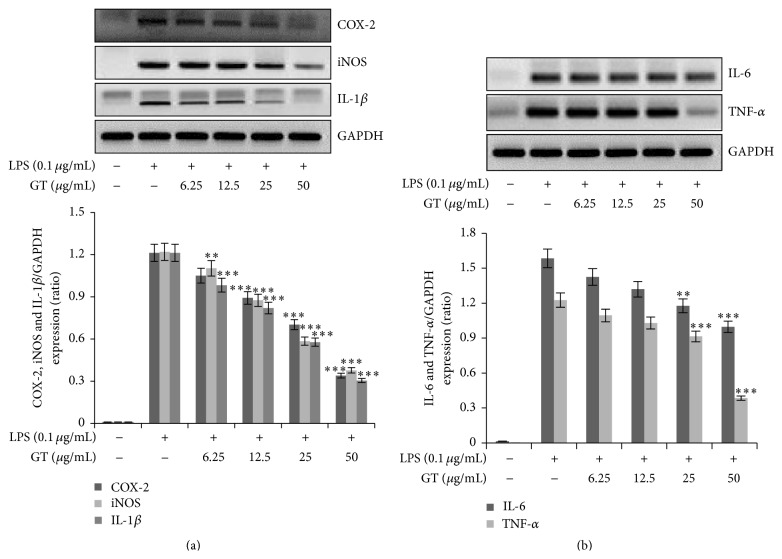
Gintonin diminished mRNA expressions of COX-2, iNOS, IL-1*β*, IL-6, and TNF-*α* in RAW 264.7 cells. For mRNA expression, RAW 264.7 cells were pretreated with gintonin (6.25 *μ*g/mL–50 *μ*g/mL) for 30 min and then stimulated with LPS (0.1 *μ*g/mL) for 18 h. Total RNA was isolated by Trizol RNA extraction reagent and mRNA expression of COX-2, iNOS, IL-1*β*, IL-6, and TNF-*α* was determined by RT-PCR. GAPDH was used as housekeeping gene. Images are representative of 4 independent experiments. Values in bar graph are mean ± SEM of 4 independent experiments. ^*∗∗∗*^ *P* < 0.001 and ^*∗∗*^ *P* < 0.005 compared to LPS only.

Proinflammatory cytokines are released as a result of inflammation induced in cell due to endotoxins. Their levels should be promptly reduced otherwise excessive release can lead to cytotoxic effects in cells. So we analyzed whether gintonin can affect the proinflammatory cytokine expression in RAW 264.7 cells. Gintonin significantly attenuated the expression of iNOS, COX-2, IL-1*β*, IL-6, and TNF-*α* mRNA expression in a dose dependent manner as has been shown in Figures 2(a) and 2(b).

### 3.3. Gintonin Inhibited the Protein Expression Levels of iNOS and COX-2

**Figure 3:**
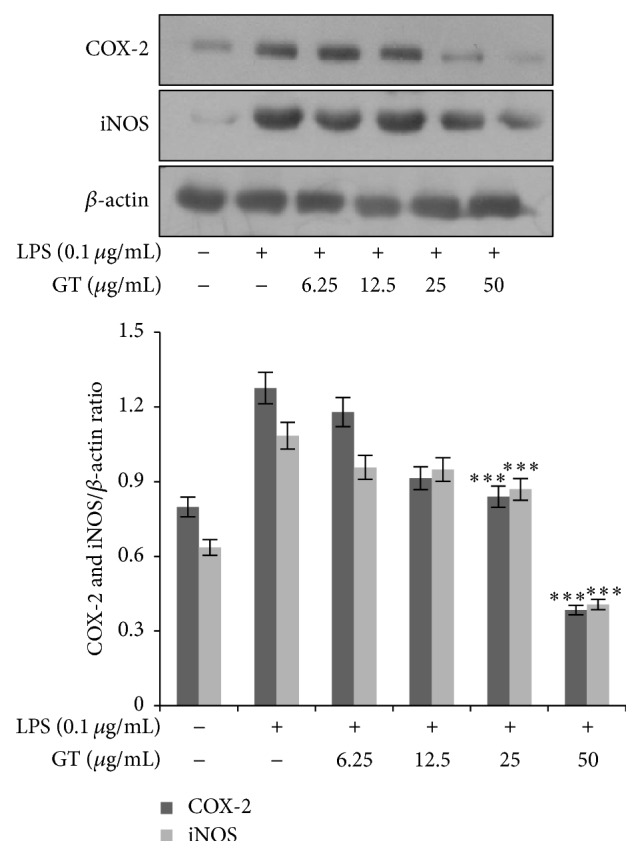
Suppressive effect of gintonin on protein expression of iNOS and COX-2. For protein expressions, RAW 264.7 cells were seeded in 6-well plate and treated with indicated concentrations of gintonin (6.25 *μ*g/mL–50 *μ*g/mL) for 30 min and then stimulated with LPS (0.1 *μ*g/mL) for 18 h. Total protein was extracted from RAW 264.7 cells using Pro-prep lysis reagent. Protein quantitation was done by Pro-Measure and then proteins were run on SDS-PAGE and analyzed by ECL chemiluminescence. Western blot images are representative of 3 independent experiments. Bar values of ^*∗∗∗*^ *P* < 0.001 compared to LPS were considered statistically significant.

To inquire if gintonin affects the protein expression levels of iNOS and COX-2, we performed immunoblotting to detect their expression. As shown in Figure 3, gintonin inhibited the protein expression of both iNOS and COX-2 in a concentration dependent manner further validating that gintonin is effective at not only transcriptional but also translational levels of these proinflammatory cytokine proteins.

### 3.4. Elucidation of Signal Transduction by Gintonin via NF-*κ*B Pathway

**Figure 4:**
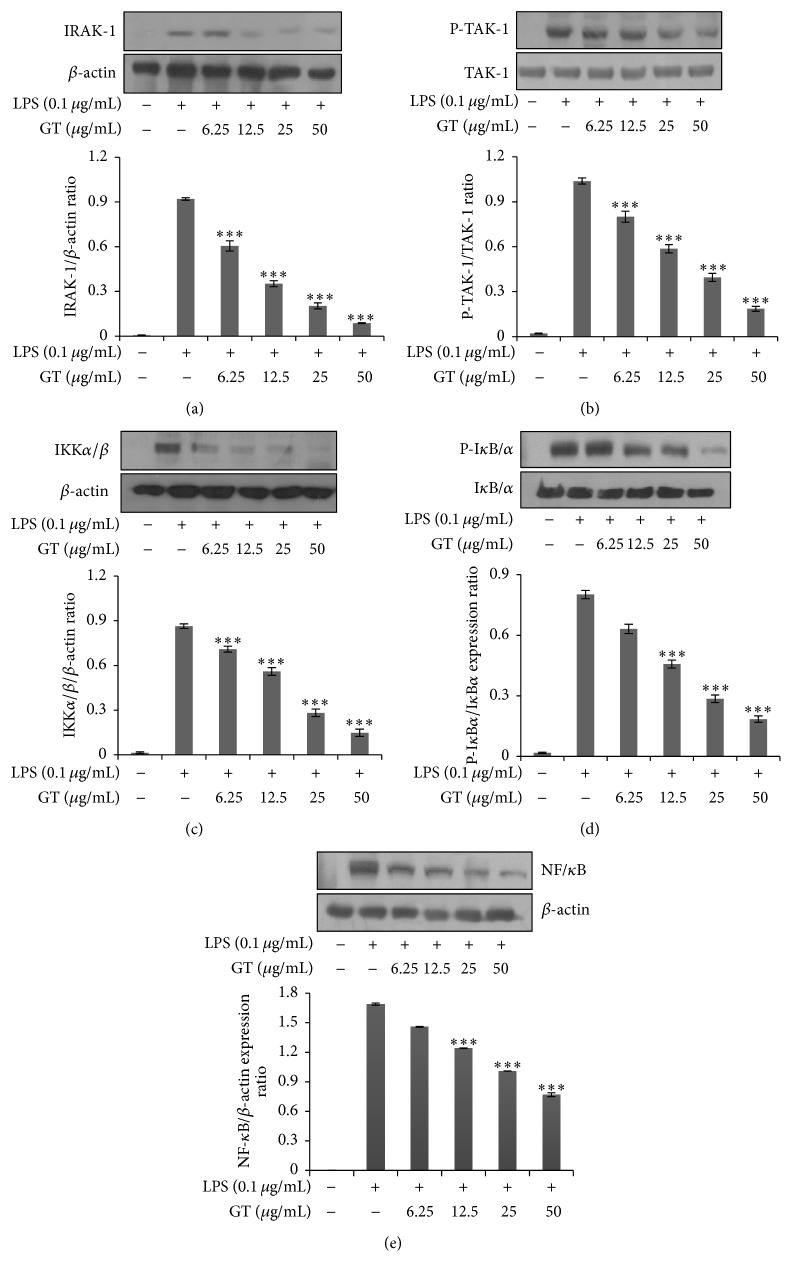
Attenuation of NF-*κ*B pathway by gintonin. RAW 264.7 cells were pretreated with gintonin in the indicated concentrations (6.25 *μ*g/mL–50 *μ*g/mL) and then stimulated with or without LPS (0.1 *μ*g/mL) for 18 h. Nuclear and cytosolic proteins were extracted using NE-PER extraction kit. Proteins were run on SDS-PAGE and treated with primary antibodies overnight followed by incubation with secondary antibodies for 2 h. The membranes were analyzed by ECL chemiluminescence systems. Data are mean ± SD (*n* = 3); ^*∗∗∗*^ *P* < 0.001 compared with LPS only. (a) Phosphorylation of IRAK-1 compared to *β*-actin. (b) Phosphorylation of P-TAK-1 compared to total TAK-1. (c) Phosphorylation of IKK *α*/*β* compared to *β*-actin. (d) Phosphorylation of P-I*κ*B*α* compared to total I*κ*B*α*. (e) Phosphorylation of NF-*κ*B compared to *β*-actin.

NF-*κ*B is one of the major canonical pathways followed by any toxin for inflammatory manifestations. Generally, this pathway is primarily activated when LPS gets attached to TLR 4 ligand. After this receptor-ligand association, a series of factors are activated. As has been shown in Figure 4(a), gintonin firstly inhibited the phosphorylation of IRAK-1 in a dose dependent manner. Afterwards in Figure 4(b), gintonin has dose dependently attenuated the phosphorylation levels of TAK-1. In Figures 4(c) and 4(d), it is shown that gintonin dose dependently diminished the phosphorylation levels of IKK *α*/*β* that caused degradation of I*κ*B/*α*. This cascade further continues in the translocation of NF-*κ*B inside nucleus that caused a strong inhibition in its phosphorylation as shown in Figure 4(e). From this result gintonin has been shown to follow classical NF-*κ*B pathway for its signal transduction.

### 3.5. Inhibition of c-Jun N-Terminal Kinase (JNK) MAPK Pathway by Gintonin

**Figure 5:**
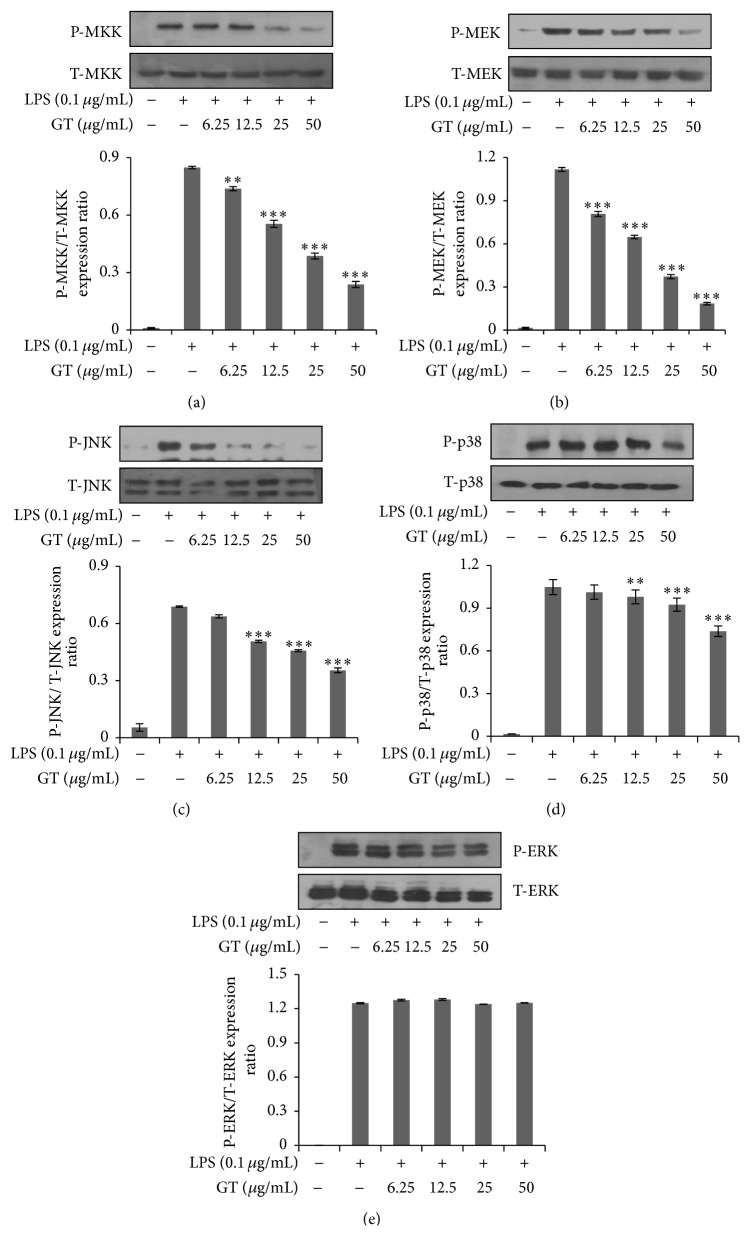
MAPK pathway inhibition by gintonin. RAW 264.7 cells seeded in 6-well plate were pretreated with gintonin (6.25 *μ*g/mL–50 *μ*g/mL) and then stimulated with or without LPS (0.1 *μ*g/mL) for 18 h. Total proteins were extracted and run on SDS-PAGE. Afterwards membranes were incubated with primary antibodies overnight followed by treatment with secondary antibody for 2 h. Proteins were then analyzed by ECL chemiluminescence system. Images are representative of at least 3 independent experiments. Data are mean ± SD (*n* = 3); ^*∗∗∗*^ *P* < 0.001 and ^*∗∗*^ *P* < 0.005 compared with LPS only. (a) Phosphorylation of P-MKK compared to T-MKK. (b) Phosphorylation of P-MEK compared to T-MEK. (c) Phosphorylation of P-JNK compared to T-JNK. (d) Phosphorylation of P-p38 compared to T-p38. (e) Phosphorylation of P-ERK compared to T-ERK.

Besides the canonical NF-*κ*B pathway, there is another strong inflammatory pathway called Mitogen Activated Protein Kinase (MAPK) pathway that also terminates into nuclear factor AP-1. Therefore, we geared to check the effects of gintonin on factors for MAPKs activation (ERK, JNK, and p38). Gintonin showed dose dependent inhibitory effect on MAPK pathway that started from the inhibition in phosphorylation of MEK and MKK as shown in Figures 5(a) and 5(b). From the dose dependent diminution in the phosphorylation of JNK and p38 as shown in Figures 5(c) and 5(d), it is clear that gintonin follows MAPK pathway as well for its signal transduction. However, it did not inhibit phosphorylation in ERK as shown in Figure 5(e). This slight deviation from regular pathway is in contradiction with the results shown by Hwang et al. [16], where gintonin suppressed ERK phosphorylation in cells expressing LPA receptors endogenously.

### 3.6. Gintonin Diminished NF-*κ*B and AP-1 Activity in HEK 293T Cells

**Figure 6:**
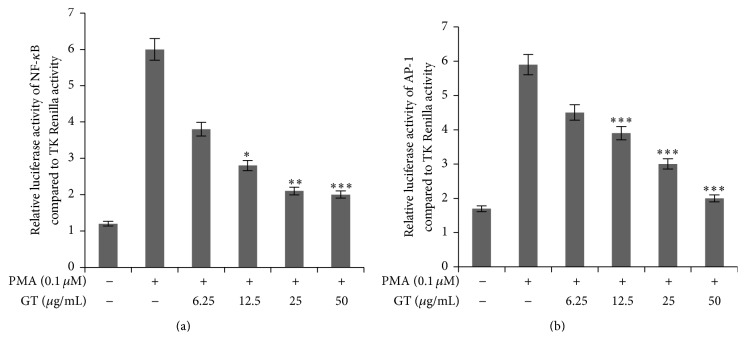
Effects on the transcriptional activity of NF-*κ*B and AP-1 by gintonin. HEK 293T cells were cultured in 24-well plate and after 18 h of incubation were transfected with NF-*κ*B and TK Renilla plasmids using Calcium-Phosphate method. 48 h after transfection, cells were pretreated with indicated concentrations of gintonin (6.25 *μ*g/mL–50 *μ*g/mL) and then stimulated with PMA (0.1 *μ*M) for 6 h. Later luciferase activity was measured using Dual-Glo luciferase assay system. NF-*κ*B activity was normalized to TK Renilla activity and concentrations were compared to PMA Renilla luciferase activity. Bar graph is ± SEM of triplicates. ^*∗∗∗*^ *P* < 0.001, ^*∗∗*^ *P* < 0.005, and ^*∗*^ *P* < 0.01 were considered statistically significant (a). Similar approach was applied for AP-1 expression (b).

For the further confirmation of signal transduction pathways followed by gintonin, HEK 293T cells were transfected with NF-*κ*B and Renilla luciferase plasmid. As shown in Figure 6(a), gintonin has dose dependently inhibited the levels of NF-*κ*B. In a similar manner, HEK 293T cells were transfected with AP-1 and TK Renilla plasmids.  Figure 6(b) shows that gintonin in a dose dependent manner suppressed the luciferase activity of AP-1 transcription factor.

### 3.7. Gintonin Recovered the Levels of mir-34a and mir-93 in LPS Stimulated RAW 264.7 Cells

**Figure 7:**
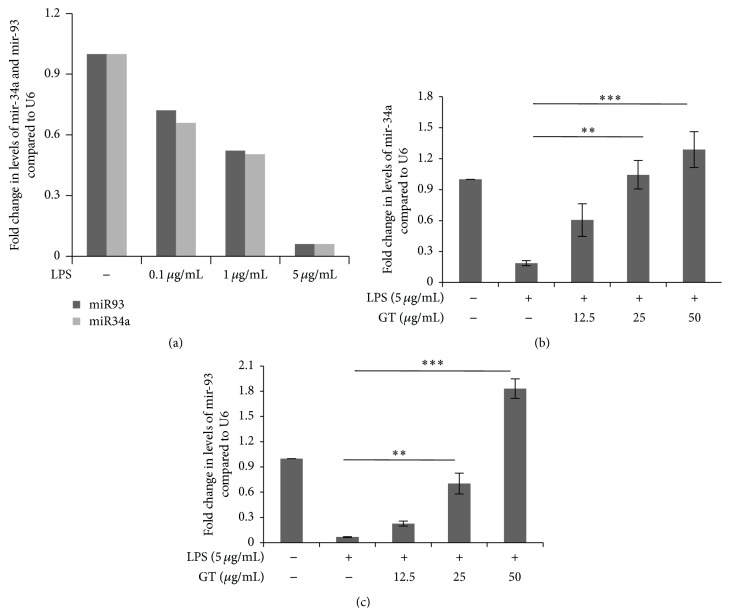
Recovery of mir-34a and mir-93 by gintonin. (a) RAW 264.7 cells were treated with LPS (0.1–5 *μ*g/mL) for 18 h. (b) RAW 264.7 cells were treated with gintonin (12.5 *μ*g/mL–50 *μ*g/mL) in indicated concentrations and then stimulated with or without LPS (5 *μ*g/mL) for 18 h. Total RNA was extracted and mRNA expression of mir-34a was observed using real-time PCR. Bar values are ± SEM of at least 3 independent experiments. ^*∗∗∗*^ *P* < 0.001 and ^*∗∗*^ *P* < 0.005 compared to LPS only. (c) RAW 264.7 cells were treated with gintonin (12.5 *μ*g/mL–50 *μ*g/mL) in indicated concentrations and then stimulated with or without LPS (5 *μ*g/mL) for 18 h. Total RNA was extracted and then mRNA expression of mir-93 was observed using real-time PCR. Bar values are ± SEM of at least 3 independent experiments. Statistic comparison is same as for mir-34a.

As has already been demonstrated by Jiang et al. and Xu et al. [17, 18], LPS downregulates the expression of mir-34a and mir-93 with increase in concentration. In relation to these findings, we pursued to check the levels of these microRNAs in LPS stimulated RAW 264.7 cells under gintonin treatment.  Figure 7(a) is in conformation with the previous results that under the LPS stimulation mir-34a and mir 93 levels are downregulated. The real-time PCR result for mir-34a showed recovery in its levels after RAW 264.7 cells were treated with gintonin (Figure 7(b)). mir-93 levels showed same result like mir-34a in presence of gintonin treatment (Figure 7(c)).

## 4. Discussion

Ginseng consists of a major portion of ginsenosides which are the saponin fractions; however, they are nonselective and require huge doses for qualification of their cellular effects [19]. After the discovery of gintonin which is a nonsaponin fraction of ginseng in 1990s, much work has been done on it in order to unravel its therapeutic potentials. Gintonin has been found to elevate calcium levels in xenopus oocytes through the activation of Gaq/11 protein couples receptors. This led to the discovery that gintonin indeed functions via lysophosphatidic (LPA) receptors which are G-protein coupled receptors (GPCRs). It has also been found efficacious for Alzheimer's disease pathogenesis as well as melanoma cell metastasis [8]. But until now the anti-inflammatory activity of gintonin remained undiscovered. In our paper therefore we have shown for the first time the anti-inflammatory activity of gintonin with special reference to microRNAs related to it.

In the second half of 19th century, Miwa et al. discovered that murine macrophages produced nitrite and nitrate in response to activation by bacterial lipopolysaccharides [20]. It is a general phenomenon that nitric oxide (NO) is a gaseous molecule that is released in response to inflammation as protective mechanism for cells. As shown in Figure 1(a), gintonin in our cell line RAW 264.7 cells has dose dependently inhibited the NO production when stimulated with LPS. We further found that gintonin did not show any cytotoxicity at all the dosage used for the study as has been observed in Figure 1(b). Cytokines are the critical players in the suppression or progression of inflammation and their levels play a key role in trauma and other inflammatory anomalies [2, 21]. In our study we found the decreasing levels of COX-2, iNOS, IL-6, IL-1*β*, and TNF-*α* by gintonin. This result indicates that gintonin potently suppresses the production of these proinflammatory cytokines, thus protecting the cells from deleterious injury.

Signaling transduction from the extracellular to intracellular potential targets is being carried out by numerous pathways. One of the very common pathways for inflammatory signal transduction is Nuclear Factor kappa B (NF-*κ*B) pathway. This pathway consists of a family of transcription factors that are very important in injury and infection. The NF-*κ*B signaling cascade begins with the activation of IKB which is in the dormant state. Phosphorylation of IKK activates I*κ*B subunit of NF-*κ*B because of which NF-*κ*B is set free and translocates into the nucleus [22, 23]. As shown in our results in Figures 4(a)–4(e), gintonin has caused a dose dependent inhibition of all the factors involved in the NF-*κ*B pathway strongly indicating that it follows the canonical NF-*κ*B pathway for its signal transduction. Proinflammatory cytokines like interleukins are released because of interleukin receptor associated receptor kinase 1 (IRAK-1) which is also responsible for activation of NF-*κ*B pathway. As shown in Figure 4(a) gintonin has also been demonstrated to decrease the phosphorylation of IRAK-1.

Besides the NF-*κ*B pathway, there is another strong inflammatory pathway followed by natural agents for the depiction of their anti-inflammatory nature, that is, Mitogen Activated Protein Kinase (MAPK) pathway. Like NF-*κ*B pathway, MAPK also consists of three distinct levels which are MAK3K, MAP2K, and MAPK. The further downstream regulators for MAPK are extracellular signal regulating kinase (ERK), c-jun-n-terminal kinase (JNK), and P38 [24, 25]. Figures 5(a)–5(d) have shown that gintonin has caused a dose dependent inhibition in phosphorylation of these MAPK factors except for ERK. Previous data showed that gintonin affected ERK phosphorylation but in our study with RAW 264.7 cells it remained undisturbed.

MKKs which belong to MAP2K are the upstream regulators for p38 and JNK and they are also being decreased by gintonin in a dose dependent manner. Other isoforms of MAP3K which are TAK1, MEKK4, ASK1/MAPKKK5, and more are also involved in the MAPK signaling pathway [26]. As can be shown from Figure 4(b), gintonin suppressed the levels of Transforming Growth Factor Beta activated Kinase 1 (TAK 1) indicating that it potently regulates its signal transduction via almost all the components of MAPK pathway because TAK-1 is a common factor for activation of both MAPK and NF-*κ*B pathways. NF-*κ*B and AP-1 are nuclear factors for NF-*κ*B and MAPK inflammatory pathways. So the luciferase assay confirmed the anti-inflammatory pathway for gintonin because it potently inhibited the transcriptional activity of NF-*κ*B and AP-1 when compared against TK Renilla activity (Figures 6(a) and 6(b)).

MicroRNAs in inflammation have already been elucidated clearly for many ailments and disease pathogenesis [27, 28]. The most common of these include mir-155, mir-146, mir-125, mir-467, mir-101, mir-132, mir-147, mir-210, and mir-92a [29–35]. However, we selected mir-34a mainly on the recent findings of Jiang et al. that demonstrated that it inhibits LPS induced inflammation in RAW 264.7 cells by targeting notch-1 [17] (Figure 7(a)). mir-34a has also been previously demonstrated for targeting stem cell marker CD44 in prostate cancer and also playing a role in NF-*κ*B pathway in esophageal cancer [36–38]. But in relation to the expression of mir-34a in natural agent like gintonin, there is almost no literature. In our research as shown in Figure 7(b), we found that gintonin potently revived the levels of mir-34a in dose dependent manner when RAW 264.7 cells were treated with 5 *μ*g/mL of LPS. It indicated that gintonin via its anti-inflammatory nature overexpresses the levels of mir-34a which has also been discovered to be an anticancerous and anti-inflammatory related MicroRNA.

mir-93 suppresses inflammation in murine macrophages treated with LPS by targeting IRAK4 as reported by Xu et al. and shown in Figure 7(a) [18]. Along with mir-34a, mir-93 is a newly discovered microRNA for inflammation. So we selected mir-93 for expression in LPS induced RAW 264.7 cells under gintonin treatment. And the results were similar to that of mir-34a expression that gintonin restored its levels in a dose dependent manner (Figure 7(c)). Mir-93 was selected in our study because of its commonalty with IRAK 4 (which is basically its target) and one of the upstream signaling factors for NF-*κ*B.

## 5. Conclusion

In a nutshell, our study demonstrates that gintonin significantly suppressed the nitric oxide levels and proinflammatory cytokines without any cytotoxicity at the given ranges. It has also been demonstrated that gintonin mediates its signal transduction mechanism via canonical NF-*κ*B and MAPK pathways but it did not affect the levels of ERK. The signal transduction pathway for gintonin was further confirmed by transcriptional repression of NF-*κ*B and AP-1 activity. The two newly discovered microRNAs (mir-34a and mir-93) were revived by gintonin indicating its anti-inflammatory nature to a micromolecular level. However, we can conclude that the therapeutic effects of ginseng cannot be attributed to the gintonin component only but it is speculated that further critical study into gintonin's pharmacologic effect will help it emerge as a functional food and novel herbal medicine for bodily anomalies.

## Figures and Tables

**Table 1:** Primer sequence used for RT-PCR for gintonin gene expression.

Gene	Primer	Oligonucleotide sequence (5′-3′)
GAPDH	F	5′-CAATGAATACGGCTACAGCAAC-3′
	R	5′-AGGGAGATGCTCAGTGTTGG-3′

iNOS	F	5′-CCCTTCCGAAGTTTCTGGCAGCAGC-3′
	R	5′-GGCTGTCAGAGCCTCGTGGCTTTGG-3′

COX-2	F	5′-TCTCAGCACCCACCCGCTCA-3′
	R	5′-GCCCCGTAGACCCTGCTCGA-3′

IL-1*β*	F	5′-CAGGGTGGGTGTGCCGTCTTTC-3′
	R	5′-TGCTTCCAAACCTTTGACCTGGGC-3′

TNF-*α*	F	5′-TTGACCTCAGCGCTGAGTTG-3′
	R	5′-CCTGTAGCCCACGTCGTAGC-3′

IL-6	F	5′-GTACTCCAGAAGACCAGAGG-3′
	R	5′-TGCTGGTGACAACCACGGCC-3′

## Abbreviations

LPA: Lysophosphatidic acid
LPS: Lipopolysaccharides
NO: Nitric oxide
iNOS: Inducible NO synthase
TNF-*α*: Tumor necrotic factor alpha
COX-2: Cyclooxygenase-2
NF-*κ*B: Nuclear Factor kappa B
MAPK: Mitogen-activated protein kinases
TLR: Toll-like receptors
IL: Interleukin
PI3K: Phosphatidylinositide 3-kinases
ERK: Extracellular signal-regulated kinases
JNK: c-Jun N-terminal kinases
IKK: Inhibitor of kappa B kinase
I*κ*B/*α*: Inhibitor kappa B-alpha
PKC: Protein kinase C
DMEM: Dulbecco's modified Eagle's medium
FBS: Fetal bovine serum
MTT: 3-(4,5-Dimethylthiazol-2-yl)-2,5-diphenyltetrazolium bromide.

## References

[1] Banerjee A., Gerondakis S. (2007). Coordinating TLR-activated signaling pathways in cells of the immune system. *Immunology and Cell Biology*.

[2] Akira S., Takeda K. (2004). Toll-like receptor signalling. *Nature Reviews Immunology*.

[3] Barton G. M., Medzhitov R. (2003). Toll-like receptor signaling pathways. *Science*.

[4] Honda K., Taniguchi T. (2006). IRFs: master regulators of signalling by Toll-like receptors and cytosolic pattern-recognition receptors. *Nature Reviews Immunology*.

[5] Hawiger J. (2001). Innate immunity and inflammation: a transcriptional paradigm. *Immunologic Research*.

[6] Bell J. K., Mullen G. E. D., Leifer C. A., Mazzoni A., Davies D. R., Segal D. M. (2003). Leucine-rich repeats and pathogen recognition in Toll-like receptors. *Trends in Immunology*.

[7] Kim Y.-J., Jeon J.-N., Jang M.-G. (2014). Ginsenoside profiles and related gene expression during foliation in panax ginseng Meyer. *Journal of Ginseng Research*.

[8] Jeong S. M., Lee J.-H., Kim S. (2004). Ginseng saponins induce store-operated calcium entry in *Xenopus* oocytes. *British Journal of Pharmacology*.

[9] Hwang S. H., Lee B.-H., Kim H.-J. (2013). Suppression of metastasis of intravenously-inoculated B16/F10 melanoma cells by the novel ginseng-derived ingredient, gintonin: involvement of autotaxin inhibition. *International Journal of Oncology*.

[10] Nah S.-Y., Kim D.-H., Rhim H. (2007). Ginsenosides: are any of them candidates for drugs acting on the central nervous system?. *CNS Drug Reviews*.

[11] Pyo M. K., Choi S.-H., Shin T.-J. (2011). A simple method for the preparation of crude gintonin from ginseng root, stem, and leaf. *Journal of Ginseng Research*.

[12] Bushati N., Cohen S. M. (2007). microRNA functions. *Annual Review of Cell and Developmental Biology*.

[13] Ambros V. (2001). microRNAs: tiny regulators with great potential. *Cell*.

[14] Bartel D. P. (2004). MicroRNAs: genomics, biogenesis, mechanism, and function. *Cell*.

[15] Choi S.-H., Shin T.-J., Lee B.-H. (2011). An edible gintonin preparation from ginseng. *Journal of Ginseng Research*.

[16] Hwang S. H., Shin T.-J., Choi S.-H. (2012). Gintonin, newly identified compounds from ginseng, is novel lysophosphatidic acids-protein complexes and activates G protein-coupled lysophosphatidic acid receptors with high affinity. *Molecules and Cells*.

[17] Jiang P., Liu R., Zheng Y. (2012). MiR-34a inhibits lipopolysaccharide-induced inflammatory response through targeting Notch1 in murine macrophages. *Experimental Cell Research*.

[18] Xu Y., Jin H., Yang X. (2014). MicroRNA-93 inhibits inflammatory cytokine production in LPS-stimulated murine macrophages by targeting IRAK4. *FEBS Letters*.

[19] Nah S. Y. (2012). Gintonin: a novel ginseng-derived ligand that targets G protein-coupled lysophosphatidic acid receptors. *Current Drug Targets*.

[20] Miwa M., Stuehr D. J., Marletta M. A., Wishnok J. S., Tannenbaum S. R. (1987). Nitrosation of amines by stimulated macrophages. *Carcinogenesis*.

[21] Dinarello C. A. (2000). Proinflammatory cytokines. *Chest*.

[22] Moynagh P. N. (2005). The NF-kappaB pathway. *Journal of Cell Science*.

[23] Perkins N. D. (2007). Integrating cell-signalling pathways with NF-*κ*B and IKK function. *Nature Reviews Molecular Cell Biology*.

[24] Plotnikov A., Zehorai E., Procaccia S., Seger R. (2011). The MAPK cascades: signaling components, nuclear roles and mechanisms of nuclear translocation. *Biochimica et Biophysica Acta—Molecular Cell Research*.

[25] Hambleton J., Weinstein S. L., Lem L., Defranco A. L. (1996). Activation of c-Jun *N*-terminal kinase in bacterial lipopolysaccharide-stimulated macrophages. *Proceedings of the National Academy of Sciences of the United States of America*.

[26] Zarubin T., Han J. (2005). Activation and signaling of the p38 MAP kinase pathway. *Cell Research*.

[27] van Rooij E., Purcell A. L., Levin A. A. (2012). Developing microRNA therapeutics. *Circulation Research*.

[28] Nahid M. A., Satoh M., Chan E. K. L. (2011). MicroRNA in TLR signaling and endotoxin tolerance. *Cellular and Molecular Immunology*.

[29] Lai L., Song Y., Liu Y. (2013). MicroRNA-92a negatively regulates toll-like receptor (TLR)-triggered inflammatory response in macrophages by targeting MKK4 kinase. *Journal of Biological Chemistry*.

[30] Lorente-Cebrián S., Mejhert N., Kulyté A. (2014). microRNAs regulate human adipocyte lipolysis: effects of miR-145 are linked to TNF-*α*. *PLoS ONE*.

[31] Qi J., Qiao Y., Wang P., Li S., Zhao W., Gao C. (2012). MicroRNA-210 negatively regulates LPS-induced production of proinflammatory cytokines by targeting NF-*κ*B1 in murine macrophages. *FEBS Letters*.

[32] Shaked I., Meerson A., Wolf Y. (2009). MicroRNA-132 potentiates cholinergic anti-inflammatory signaling by targeting acetylcholinesterase. *Immunity*.

[33] Tian G.-P., Chen W.-J., He P.-P. (2012). MicroRNA-467b targets LPL gene in RAW 264.7 macrophages and attenuates lipid accumulation and proinflammatory cytokine secretion. *Biochimie*.

[34] Wei Y., Nazari-Jahantigh M., Neth P., Weber C., Schober A. (2013). MicroRNA-126, -145, and -155: a therapeutic triad in atherosclerosis?. *Arteriosclerosis, Thrombosis, and Vascular Biology*.

[35] Zhu Q.-Y., Liu Q., Chen J.-X., Lan K., Ge B.-X. (2010). MicroRNA-101 targets MAPK phosphatase-1 to regulate the activation of MAPKs in macrophages. *The Journal of Immunology*.

[36] Fujita Y., Kojima K., Hamada N. (2008). Effects of miR-34a on cell growth and chemoresistance in prostate cancer PC3 cells. *Biochemical and Biophysical Research Communications*.

[37] Li Y., Guessous F., Zhang Y. (2009). MicroRNA-34a inhibits glioblastoma growth by targeting multiple oncogenes. *Cancer Research*.

[38] Nalls D., Tang S.-N., Rodova M., Srivastava R. K., Shankar S. (2011). Targeting epigenetic regulation of mir-34a for treatment of pancreatic cancer by inhibition of pancreatic cancer stem cells. *PLoS ONE*.

